# Design, Fabrication, and Characterization of Magnetoresponsive Materials and Devices

**DOI:** 10.3390/ma15207183

**Published:** 2022-10-14

**Authors:** Karla J. Merazzo

**Affiliations:** 1 BCMaterials, Basque Center for Materials, Applications and Nanostructures, UPV/EHU Science Park, 48940 Leioa, Spain; karla.jaimes@ucr.ac.cr or karla.merazzo@bcmaterials.net; 2 Materials Science and Engineering Research Center (CICIMA), Universidad de Costa Rica, San Pedro 11501-2060, Costa Rica; 3 Escuela de Física, Universidad de Costa Rica, San Pedro 11501-2060, Costa Rica

Modern technology has made an elegant link between smart materials and interlinked devices thanks to the interplay between materials science, smart sensors and devices, artificial intelligence, and a fierce imagination; this has allowed us to reach every corner of our society. Smart or responsive materials are ones that respond in a controllable manner to an external stimulus by changing one or more of their properties. Some external stimuli include the following: temperature, stress, electric, chemical, and light. 

Among these, we have the magnetoresponsive materials and devices, which are materials that respond to an external magnetic field; these are considered as “*an exclusive class of smart materials that are highly valuable due to their magnetically activated smart and/or multifunctional response*” [1]. They are the part of the new industrial revolution, allowing improvements in the quality of life with respect to biomedical applications [2]; they bring new robotics solutions to life [3]; they increase the number of sensors and actuators [4,5] and provide better answers to our environmental issues [6], among many others. All this provides opportunities to push our limits in whatever endeavour we propose.

The great advantage that characterized the magnetoresponsive materials is contactless activation, their fast switching (in some cases), and their versatile ability with respect to applications in a myriad of devices made by using different fabrication techniques, thanks to its ability to adapt to different needs and applications (such as composites by additive manufacturing [1], polymeric composites [7], nanofabrication by lithography techniques [8], and by using microfluidic devices [9], among others).

In this Special Issue, new studies on magnetoresponsive materials and devices will be presented to widen our understanding of this topic, and all of them have different applications, such as magneto-active energy; magneto-active memory-storage; magneto-active sensors; magneto-active actuators; magneto-active material for biomedical applications and devices; and the advanced manufacture of magnetoresponsive materials and devices. This Special Issue welcomes different fabrication techniques for obtaining magnetoresponsive materials and devices, their wide range of applications, their characterization, theoretical works, and all innovative results.
